# Perspectives and Practices on Water, Sanitation, and Hygiene from a Fishing Community along Lake Malombe, Southern Malawi

**DOI:** 10.3390/ijerph17186703

**Published:** 2020-09-15

**Authors:** Limbani R. Kalumbi, Chisomo Thaulo, Eleanor E. MacPherson, Tracy Morse

**Affiliations:** 1 Department of Environmental Health, University of Malawi—The Polytechnic, Private Bag 303, Chichiri, Blantyre 31225, Malawi; cthaulo@poly.ac.mw; 2 Centre for Water, Sanitation, Health and Appropriate Technology Development (WASHTED), University of Malawi—The Polytechnic, Private Bag 303, Chichiri, Blantyre 31225, Malawi; tracy.thomson@strath.ac.uk; 3 Malawi Liverpool Wellcome Trust, Queen Elizabeth Central Hospital, College of Medicine, P.O. Box 30096, Chichiri, Blantyre 31225, Malawi; Eleanor.MacPherson@lstmed.ac.uk; 4 Department of Civil and Environmental Engineering, University of Strathclyde, Level 5 James Weir Building, Glasgow G1 1XQ, UK

**Keywords:** fishing community, WASH, Malawi, marginalised population

## Abstract

People living in fishing communities have a high burden of preventable water, sanitation, and hygiene (WASH) related diseases but have often been neglected in research and policy. We explored practices and perspectives on WASH among fishing villages around Lake Malombe, Malawi. We employed a mixed methods design, and data were initially collected through participant observations (five weeks), followed by a second phase of qualitative interviews (*n* = 16), focus group discussions (*n* = 7), and quantitative surveys (*n* = 242). We observed that safe water sources were scarce; latrines were basic; and handwashing facilities were limited. Seventy-one percent (*n* = 174) of households collected water from unsafe sources (open wells and the lake). Eighty-six percent (*n* = 207) of households had basic short-term latrines. Twenty-four percent (*n* = 59) of households had handwashing facilities with soap. Qualitative data supported these observations and identified additional factors which compounded poor WASH practices including, a high transient population associated with the fishing trade, poor infrastructure design and construction which lacked consideration of the environmental factors, context and social and cultural norms. As such, fishing communities are underserved and marginalised with constrained access to WASH services, which must be addressed through behaviour-centered and context appropriate solutions.

## 1. Introduction

Despite an estimated 107 million people living around the African Great Lakes [1], there is a paucity of health related research and interventions in fishing communities. Their neglect is often attributed to the fact that they can be geographically remote [1,2]. Fishing communities have been recognised as being amongst the most vulnerable, poor, and marginalised populations in low income countries [3,4], despite being an integral component of the economy and food security. Research has shown that communities close to water bodies are at risk of multiple infectious diseases as a result of water interactions, including water-borne and water-related diseases, and neglected tropical diseases (human schistosomiasis and soil transmitted helminths). Further, they have also been identified as being more vulnerable to HIV and AIDS than the general population [5]. In Malawi and Zambia, fishing communities have been found to be more at risk from cholera, diarrhoea, and dysentery owing to poor water quality and general low hygiene standards [2,6,7].

Evidence has shown that access to improved sanitation can reduce more than a third of diarrhoeal disease [8], and can drastically minimize the adverse health impacts of other disorders responsible for morbidity and mortality. The availability of water for personal and domestic hygiene has been found to be an important factor in decreasing the rates of water-related diseases such as ascariasis, diarrhoea, schistosomiasis, and trachoma [9]. For instance, a recent study in South Africa found that the provision of safe water contributed to an eight-fold decrease in children’s risk of urogenital schistosomiasis infection [10]. Risk factors contributing to periodic diarrhoea and cholera outbreaks in fishing populations have included: Lack of access to sanitary facilities and potable water for fishermen while fishing, overcrowding on the beaches, poor water quality, low levels of hygiene, and poor waste management [2,11,12]. These problems are compounded by the lack of sanitation and hygiene facilities in these populations [2,12,13].

Notwithstanding population health impacts, safe water, hygiene practices, and hygienic production processes are similarly vital to ensure that fresh fish and fish products, a key source of income and food security, are wholesome [14,15]. Care in fish handling is paramount, particularly between capture, processing, and vending of fish, because the quality, safety, and shelf life of the products are dependent on proper handling and processing practices [12,14,15]. Studies in Malawi have shown that there is a high risk for microbial contamination at all stages from capture to sale of fresh fish from artisanal fishermen, and this was significant across all seasons [12]. The presence of *E. coli* in this study highlighted the risk of faecal contamination, which could be related to personal hygiene practices. This underlines the public health concern of poor hygiene management of fish and fish products, including dried and smoked ready to eat products, sold in local markets [16,17].

Noticeably, the scarcity of safe drinking water and inadequate sanitation exacerbated by poor hygiene practices places fishing communities at an increased risk of diseases, and ultimately, impacts on well-being [2,11,12]. As such, fishing communities are an important sector of the population to consider in terms of water, sanitation, and hygiene (WASH) services, and small-scale fishing communities have often been neglected in policy as well as public services [18]. As neglected communities, there continues to be a dearth of information on the status and practices of WASH among fishing communities. We aimed to address this knowledge gap and explore in depth, the practices, perspectives and status of water, sanitation, and hygiene among communities around Lake Malombe. Understanding these practices, and norms and attitudes that perpetuate them, can be used to introduce effective interventions in the future.

## 2. Materials and Methods

### 2.1. Study Setting

**Figure 1:**
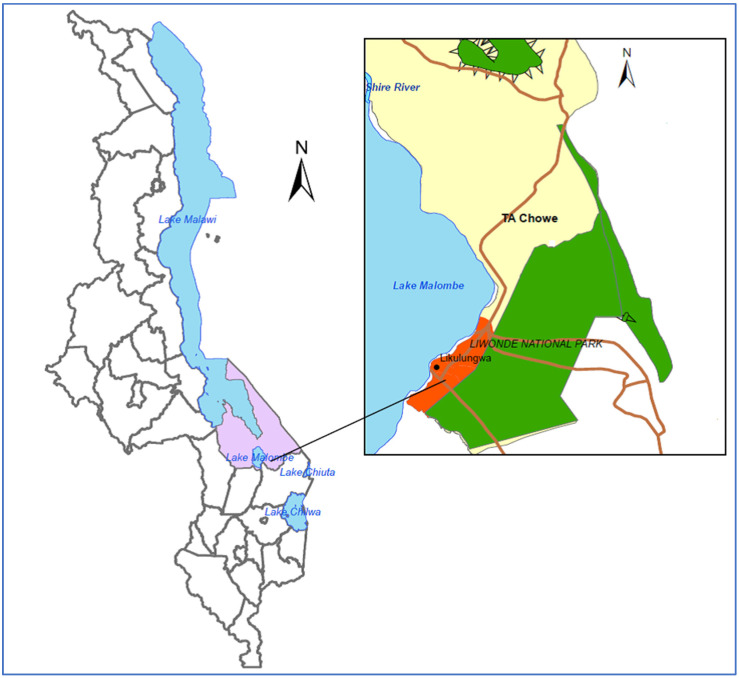
Map showing Mangochi District and the study area.

The study was conducted in Mangochi District, which is situated in the Southern Region of Malawi. The District has two lakes: Malawi and Malombe. The work was situated in fishing communities along Lake Malombe. The lake is estimated to support over 2205 full-time fishermen [19]. With guidance from the District Health Office we selected Likulungwa, a hard-to-reach low-income fishing community located on the east bank of the lake (Figure 1). The community had seven villages with approximately 630 permanent households in total with an estimated population of 2835. However, this populace was highly dynamic due to its additional transient population, with high mobility and migration of non-resident fishermen and fish traders. Fishermen migrate from one part of the country to another throughout the year in search of fish catches.

### 2.2. Study Design

The study employed a mixed methods approach, utilising both quantitative and qualitative methods. The design was a hybrid sequential exploratory mixed method (QUAL → quant + QUAL). Data were collected in two phases. Phase one involved participant observations (PO) for a period of five weeks; the results of this first phase informed the subsequent phase which involved in-depth interviews (IDIs), focus group discussions (FGDs), and a quantitative survey.

### 2.3. Phase One (October to November 2018)

Participant observations were conducted for a period of five weeks by a team of two researchers (male and female). The research team lived in the study community observing all aspects related to fishing, livelihoods, and specifically WASH. The observations included the following aspects: Day to day life of fishermen, fish processors, fish sellers and community members; landing of fishing boats; processing of fish in sheds and households; selling of fish; and opportunities for targeted WASH behaviours and practices in the households, public places, and at the lake. As part of observations, in order to build up a fuller picture of WASH practices and dynamics of the communities, informal discussions were employed with community members and potential research participants. During observations, brief notes were taken and then expanded on in the evenings.

### 2.4. Microbiological Analysis of Drinking Water (November 2018)

After participant observations, we collected a total of ten water samples from boreholes (*n* = 3), unprotected wells (*n* = 6), and Lake Malombe (*n* = 1). The samples were analysed for the presence of *E. coli*. Water samples were collected in sterilised screw-top Fischer bottles and were stored in a cooler box. Within three hours all samples were received at the Polytechnic laboratory and processed immediately. Samples from each water source were tested using IDEXX Colilert-18^®^/Quanti-Tray 2000 (IDEXX Laboratories Inc., Westbrook, ME, USA) defined substrate methods to determine the most probable number (MPN) for both coliforms and *E. coli*. As per IDEXX procedures we added a chemical substrate—which contains 4-methyl-umbelliferyl β-D-glucuronide (MUG)—to a 100-mL sample and mixed. The mixture was poured into a multi-well tray, and sealed. The sealed mixture was incubated for 18 to 24 h at 37 °C, and the samples were examined for colour change (coliforms) and fluorescence (*E. coli*). The standard MPN table was used to convert the number of wells that fluoresced to MPN/100 mL.

### 2.5. Phase Two (December 2018)

#### 2.5.1. Qualitative Component

For the second phase of qualitative research a purposive sample was used based on a sampling frame developed from insights in phase one. We sampled based on involvement in the fishing industry, gender, age, and vulnerability to select participants who were information rich. Overall, 16 IDIs were conducted face-to-face with the following categories of people: Fishermen (*n* = 2), male and female fish processors (*n* = 4), fish helpers (*n* = 2), boat owners (*n* = 2), transient population (*n* = 2), businessperson (*n* = 1), farmer (*n* = 1), and vulnerable groups (elderly) (*n* = 2). A total of seven FGDs were conducted with the following groups: Community members (men and women separated based on gender) (*n* = 2), fishermen (*n* = 2), fish processors (*n* = 1), and male and female youth (*n* = 2). For IDIs and FGDs, semi-structured interview guides—informed by phase one—were used to collect data. IDIs and FGDs targeting women were conducted by a female research assistant, and those targeting men by a male research assistant. On average, IDIs and FGDs lasted approximately 45–90 min, all were audio recorded, transcribed, and translated verbatim from the local language (Chichewa) to English.

#### 2.5.2. Quantitative Method

The quantitative survey employed a probability sampling method, using a proportional allocation of households per village based on total village household population relative to the entire study population. Using a list of households from the Village Head, we randomly selected households using the Microsoft Excel software. A random sample of 242 households from across the seven villages participated in the survey. The household head, or an adult member in the absence of the head, was interviewed using a structured questionnaire. Questions addressed demographics, access, and use of WASH services.

### 2.6. Data Analysis

Field notes from participant observations and transcripts from FGDs and IDIs were checked against audio files. Qualitative data were analysed using thematic analysis guided by a framework approach [20]. The principal investigator and another researcher independently coded transcripts line by line and generated codes based on emergent themes. Codes were reviewed and patterns of agreement and discrepancy established were deliberated by the two researchers (L.R.K. and C.T.). Field notes from the observation were also used during data collection and analysis. Quantitative data were analysed using Stata 14.1 (StataCorp, 2015, College Station, TX, USA). In this study, we represent descriptive statistics as means and standard deviations for continuous variables and proportions for categorical variables, significant differences were assessed through Chi-square tests and the association of dichotomous outcomes—for example, access to safe water (safe/not safe)—and covariates have been estimated through prevalence ratios (PR) and are reported with 95% confidence interval. The study integrated qualitative and quantitative data at reporting and interpretation stages through narratives.

### 2.7. Ethical Considerations

This study obtained the approval of the National Committee on Research in the Social Sciences and Humanities (NCRSH) Protocol no. P.07/18/293. In the field, data were collected with the informed written consent of study participants prior to their participation.

## 3. Results

### 3.1. Demographics

Within household surveys, the majority of respondents were female (65.7%). The age range of the respondents was 18 to 79 with mean of age 38.2 (SD = 15.3). Just over half of participants (54.6%) had primary education, and noticeably, 42.6% had never attended school. The majority of respondents (80.2%) were married and 55.8% were Muslim. Respondents were predominantly Yao ethnicity (57.4%) and most (63.6%) were farmers. Farming (63.6%) was the dominant livelihood, and 8.3% of respondents were involved in the small-scale fishing industry. The smaller proportion involved in the fish industry could be attributed to a shift in livelihoods due to declining fish stocks, and is indicative of fishing being the primary livelihood of transient rather than permanent populations.

### 3.2. Water

#### 3.2.1. Access to Water

We observed a persistent lack of potable drinking water across all the study villages, with “safe water” being largely obtained from three functioning communal boreholes. However, 69% of households reported having insufficient quantities or unacceptable drinking water; reasons included: Water not available from the source (44.1%), low yield (25.6%), source broken down (10.7%), congestion (10.1%), source not accessible (8.3%), and water too expensive (1.2%). As such, some households had dug unprotected wells in order to increase water access—we recorded six wells. From household surveys, 71.9% collected drinking water from unsafe sources: Unprotected wells and lake water (Table 1). Seventy one percent of households reported not paying to access water, and 22.3% reported paying monthly; on average households contribute MK 200.00 ($0.28) per month. There was a significant association between payment for water, and use of a safe water source (*p* < 0.0001); the frequency of paying for water services was 17 times greater in households who reported using safe water sources (boreholes) than in households using unsafe sources (PR = 17.3, CI: 8.6–34.9). Qualitative findings corroborated the lack of sufficient water sources. Invariably in all interviews, the challenges of accessing safe drinking were raised, and although wells were available (sometimes in a walkable distance) these were deemed as an unsatisfactory alternative due to water being dirty or unsafe.

“*Aaaa here, the main problem we face is water. If you would walk to that side [pointing to a distance of almost 200 m], you can see a well which we use. We seriously face problems with water*”.

*(42-year-old businessman, IDI)*

The majority (75.2%) of households reported requiring more than 30 min to collect drinking water (Table 1) due to distance or congestion, placing a heavy burden on those responsible. Less than half (47.9%) of the participants reported fetching water at a distance of less than 500 m. Interestingly, those who reported using unsafe sources travelled distances greater than 500 m compared to respondents who used safe sources (*p* < 0.0001), primarily to avoid congestion, and find water which was not salty. During interviews, participants felt that the return time for fetching water was long, and time taken to gather water had a significant impact on their lives and livelihoods. Further, the settings in Malawi and fishing communities are significantly gendered, and within the fishing industry, women undertake selling and processing of fish; therefore, spending more time accessing water impacts women’s participation in livelihood and household activities.

“*If we had more boreholes in this community I think people can never draw water from the wells, but this is happening because of the lack of boreholes in this community. Because when you go to the borehole you find young girls fighting to draw from the borehole, so we just go to the wells as women. We even go to fields late because we spend a lot of time fetching water which also affects our field work*”.

*(37-year-old woman, FGD)*

#### 3.2.2. Perception of Quality and Safety of Drinking Water

In terms of perceived quality of the water, participants felt that water from the unprotected wells was visibly dirty, however was more acceptable than water from the boreholes which was “salty”. Community members described, how salty water from boreholes shaped their decision to use unprotected wells. During informal discussions women described how salty water presented multiple challenges beyond unpleasant taste and included: Soap not producing enough lather when washing; and legumes taking a prolonged period to cook, which impact on the cost or the need for more firewood. We observed that drawing of drinking water was not necessarily about a single preference, but rather a combination of factors which drove people to collect water from unsafe sources. This is highlighted in the quotes below:

“*The water from boreholes is salty. It tastes like you have added soda and salt in the same water. So, legumes don’t get cooked. All the relish that we get from a tree get hard to get cooked*”.

*(40-year-old woman, FGD)*

“*It’s not about preference, it’s about getting the water and using it. The water from the borehole is salty while the water from the wells is not safe: debris, dirty, soil can all fall in when it’s windy and make the water dirty. If you can see it, you can’t even drink it. We are surviving because of God*”.

*(24-year-old fisherman, IDI)*

Based on the participant observations around different access points we decided to measure the microbiological quality of the sources in the community based on quantification of *E. coli*. The Malawi Bureau of Standards through the Malawi Standards (MS) 733 drinking water specification for boreholes and shallow wells recommends that *E.coli* should be absent in all drinking water [21]. Only water from two boreholes met acceptable standards (Table 2). This concurred with household perceptions during quantitative surveys, where nearly half (47.1%) perceived that the water was not safe (Table 1). Not surprisingly, those who used safe water sources perceived that water was safe for drinking compared to those who used unsafe sources (*p* < 0.0001). The proportion of respondents using safe water sources was 4-fold greater if they perceived that their drinking water was safe (PR = 4.7, CI: 2.4–8.9).

#### 3.2.3. Treatment of Drinking Water and Storage

Although the community was aware that the water they drink is unsafe, and were knowledgeable of simple water treatment techniques such as chlorination and boiling, these techniques were infrequently used and were never observed. Key factors cited as shaping this behaviour included: Lack of money to buy water treatment materials, the bad smell of water in a case of chlorine, and that the practice was labour intensive and time consuming. Participants described how chlorine was intermittently distributed in the community by health officials, but that concerns over chlorine causing illness and headaches meant uptake was infrequent. Instead, chlorine was used for other purposes including the treatment of smell from pit latrines. This discrepancy between knowledge and action demonstrated that knowledge of risk did not translate to deliberate risk averting behaviours such as water treatment, instead, this knowledge was outweighed by aesthetic factors (taste and smell) and usage factors (cooking and drinking).

“*We can say almost everyone here does not use the chlorine that we are given. Because of the smell, most people do not like to use it in the water and we don’t get ill. People complain about headaches due to the smell of the chlorine, and most people just use it to kill smell in the latrines*”.

*(50-year-old woman, FGD)*

Although the household survey showed that 40.1% of households reported that they treated drinking water (Table 1), when compared to the qualitative findings there are indications of overreported behaviour. There was no difference in the reported treatment of drinking water between those who accessed safe water sources (38.2%, 95% CI: 26.8–50.8%) and those who did not (40.2%, 95% CI: 32.9–47.9%). As regards to the treatment method: 77.1% had used chlorine, 13.5% had boiled, and 4.2% had strained through a cloth at some time, with only 11.5% reporting that they always practiced the behaviour (Table 1). There were significant differences between those who treated drinking water using the boiling method and those who used chlorine (*p*-value = 0.003); the proportion of respondents reporting to always treat drinking water was 7-fold greater if respondents reported the boiling method compared to using chlorine (PR = 7.1, CI: 2.2–23.1). Only 4% of the households had a water storage container which was narrow-mouthed, and 87% of water storage containers were covered with a lid. The majority (97%) of households accessed water through dipping, a practice which increases the risk of contaminating drinking water.

#### 3.2.4. Beliefs Associated with Water

We observed existing beliefs associated with water. In one incident, when one of the observers went to draw water from one of the wells, a young girl—probably aged 13 or 14—found a frog in the bucket used to draw water from the well, she immediately threw the frog back into the well. Upon being asked the significance of doing that, she asserted that their mothers taught them that the frog brings more water to the well. This experience demonstrated that the community’s handling of water was anchored in their cultural beliefs and practices; consequently, their interaction with water ensured maintaining congruence with the elements of these beliefs. Another belief was that lake water away from the shore was safe, since it is a large water body. As such, drinking lake water abstracted a few meters from the shore was more acceptable. This belief was equally shared by community members and fishermen, regardless of the well-known practice that fishermen use the same lake at night for defaecation while conducting their business, a practice which potentially contaminates the water they perceived as safe. These misconceptions are being perpetuated from generation to generation and shape existing practices in terms of using water.

### 3.3. Sanitation

#### 3.3.1. Latrine Access and Design

The majority of the households had latrines (86%), however, the majority (85%) were very basic in construction (Table 3); latrine floor was made of tree logs covered with soil, superstructures were largely made of thatch, and most did not have a roof, where roofs were present, they were made of thatch. IDI and FGD participants reported that while most households have latrines, rooms (rest houses) that accommodate transient fishermen, processors, and buyers did not have enough latrines. This was due to unwillingness of the owners to construct them, lack of materials, cost, and sandy soils leading to latrine collapse.

“*The thing is these people stay at the rooms when they come in this community to buy and process fish. So, the rooms lack good care. There are no enough latrines in the rooms*”.

*(50-year-old woman, FGD)*

We observed inadequate sanitation facilities at the rooms near the shore tended to encourage latrine sharing, in some cases one bathroom and toilet would be used by seven to ten rooms. Furthermore, the presence of transient fishermen, processors, and buyers intensified this practice. From household surveys, the majority (64.7%) reported sharing their sanitation facility; mostly with neighbours (34.3%) and general public (14.9%).

We observed that the toilets were small compared to a standard latrine, one of the observers who visited the latrines noted: *“when I stood up my head touched the roof”.* IDI and FGD participants reported that most of the latrines in the community were basic. In keeping with Muslim traditions, we observed that most latrine designs had a bathroom attached, likely to aid in anal cleansing; and during the survey, we observed that 73.9% of the latrines had anal cleansing materials—a bucket or clay pot containing water, but rarely soap. Similar to latrines, the bathrooms were mostly made of thatch and had no roof. We observed that the design of these sanitation facilities—where the front part was the bathroom and the rear was the toilet itself—slightly improved privacy, though, doors were mostly makeshift worn out pieces of cloth. These findings were supported during the household survey, in terms of privacy, only 1.9% of the latrines were lockable inside, and 1.4% of the facilities were lockable outside to deter use by non-household members (Table 3). Further, 44% of the latrines had no drop hole covers whose absence increases the chances of faecal-oral transmission of diseases, as flies have free access to faeces which can be deposited on food and fluids.

We observed, and community members conceded, that it is hard for the people living near the lake to construct stable latrines due to sandy soils. Most households (66.7%) with latrines reported that they have experienced collapse. The reasons cited for collapse included: Flash floods (48.6%), poor construction materials (39.1%), and weak design (11.6%). Upon being asked if collapse prompted any change to the future latrine design, 51.5% reported constructing a more stable latrine, whereas 47.8% did not change anything. Key themes from the qualitative analysis outlined cost and lack of knowledge on proper latrine designs fitting their context as reasons for lack of change.

“*When the roof is well covered with a plastic paper, it can withstand the rains and the winds. It only collapses when the water flashes from the lake to the villages*”.

*(24-year-old fisherman, IDI)*

#### 3.3.2. Defaecation Practices of the Community

During observations participants said the practice of households owning and defaecating in a latrine was a contemporary experience in this fishing community. Previously, the lake and surrounding bushes were the main areas where defaecation took place, and we observed vestiges of continuing open defaecation. For instance, in one incident, while mending nets, human faeces were a few meters from where the net repairers were stationed. From household surveys, among those without latrines (*n* = 35), 94.3% indicated using the neighbour’s latrine, whereas 5.7% indicated using the lake and bush. Therefore, self-reported open defaecation (OD) rate was low (0.8%) but may have been prompted by reflexivity in response.

“*Aaah, I defaecate in the latrine at my compound. There are many latrines in this community unlike previous years. Maybe five or four households have latrines for every 10 households*”.

*(50-year-old fisherman, IDI)*

Data from IDI and FGDs indicted a long-entrenched practice of open defaecation in this fishing community; and this resulted in indifferent attitudes towards OD. Study participants agreed that there were OD cases in the area, and in some cases they find faeces at the back of their households, which they attributed to transient fishermen, processors, and buyers.

“*And some people may even come at night and relieve (defaecate) at the back of your house. These problems are coming because of these transient fishermen and buyers because they do not have latrines*”.

*(36-year-old woman, FGD)*

In addition, the absence of public toilets at the shore intensified the practice of OD, this is highlighted in the following quote:

“*We lack latrines here at the lake, as such we opt to defaecate in the water which leads to diarrhoea and cholera outbreaks*”.

*(48-year-old net repairer, FGD)*

We observed little regard of child faeces, in most cases caregivers delayed removing the faeces from the ground or nappy cloth, which were later thrown in nearby fields, rubbish pits, and in latrines. The delay was mediated by other pressing issues such as household chores and the perception that child faeces were less harmful. During FGDs, participants described different ages when child faeces were considered harmful; they agreed that child faeces were only harmful when the child starts complementary feeding. These practices and perceptions resulted in longer periods of possible contact between vectors (flies) and faeces, thereby increasing the risk of disease transmission.

“*We take the child from where she has defaecated and remove the faeces and throw them into the latrine. If there is water we wash what the child was wearing and throw the water into the toilet not anywhere else. If there is no water we put the clothes in a plastic bag and wash at the lake*”.

*(38-year-old woman, FGD)*

#### 3.3.3. Sanitation Practices of Fishermen, Processors, and Buyers

As their work is mostly done at night and far from the lake shore—now that catches are declining they have to push much further for a substantial catch—sanitation options for fishermen are very restricted. As a result, fishermen usually use the lake for urination and defaecation when carrying out their work. They recognised this as a major challenge they have, and additionally, the fishermen felt that this practice was undignified. One fisherman said:

“*Not that we are comfortable, but that’s the only place we have to defaecate when fishing. Where else would we go? The good thing is that the water is not stationary and the faeces become fish food. When deafecating on the lake, there’s no self-respect or dignity there. You defaecate when everyone else is there watching you. We don’t feel comfortable, but as time goes we just give in to this kind of life*”.

*(24-year-old fisherman, IDI)*

We observed that this practice was recognised within the entire community, and has been normalised as an acceptable practice cognizant of the limited options they have. Fishermen, transient population, and community members also believe that fish eat the faeces and the lake self-cleanses.

### 3.4. Hygiene

#### 3.4.1. Presence of Handwashing Facilities

Visual inspection of surveyed households showed that 61.6% had a handwashing facility. The common type of handwashing facility was a mobile facility (basin and jug used in multiple locations) (82.6%), 17.4% had a fixed handwashing facility (HWF) within the plot/yard. Respondents were asked to show where they wash hands after defaecation, 46.3% (*n* = 112) showed the same location where they frequently wash hands, and a significant proportion (50.0%) reported that they do not wash hands after defaecation. Similarly, 55.4% of the households pointed to the location for handwashing before preparing food, and a significant proportion (42.2%) reported that they do not wash hands before food preparation. Water was available in handwashing facilities of 59.7% households. Soap was present on HWFs of 24.3% of the total surveyed households.

During FGDs and IDIs, nearly all the participants understood the importance of handwashing. Despite this, participants reported that few households had fixed handwashing facilities (tippy tap) and handwashing materials such as soap were scarce due to lack of money.

“*Handwashing is important to remove germs from our hands but we just wash our hands with water only at the bathroom because we do not manage to get soap for handwashing. If we can’t manage to get soap for bathing, how can we manage to get soap for handwashing?*”.

*(75-year-old woman, IDI)*

The presence of a handwashing facility outside the latrines clashed with the majority of the community’s religious beliefs. An appreciable majority (55.8%) of the community were Muslim and they practiced anal cleansing—done with water and soap (if available) in the latrine or in bathrooms connected to latrines, 27% of the surveyed Muslim households had soap available for anal cleansing or handwashing. This was deemed adequate, and they felt no need of another handwashing as that would mean “repeating handwashing”, hence rendered redundant.

“*Muslims conduct anal cleansing when they are leaving the toilet after defaecating because the toilet is very close to the bathroom. And that’s the only time I know they wash their hands*”.

*(51-year-old fisherman, IDI)*

#### 3.4.2. Handwashing Behaviour

We observed that handwashing with soap was a rare event and was witnessed only when women’s or caregiver’s hands were visibly soiled from garden work, household chores, and if there were guests to be served. There was no attempt to encourage children to wash hands after visiting the toilet or before eating. We viewed numerous occasions when children’s hands were dirty while eating food in full view of adults who looked unaware or unconcerned of the practice. Although some households have constructed hand washing facilities under the direction of government extension workers and non-governmental organisations (NGOs), these are not in common use. During informal discussions one woman said: *“Hand washing facilities are good, but then most of the people do not use them. They just put them for the sake of making the government happy”.*

We witnessed handwashing being commonly practiced, albeit without soap, during lunch or supper. The shared practice of dipping hands into a common basin containing water was prevalent; similar practices were observed among fish processors. The practice of handwashing after defaecation was also an infrequent event, most latrines had anal cleansing materials stationed inside the latrine or in the bathroom. After defecation, community members recounted using water and soap (if available) to clean their bottoms.

We found that the practice of handwashing is anchored in beliefs that have been passed down from generation to generation. Women reported that infants and young children are given water to drink that has been used for washing hands of adults to promote growth. A practice such as this one is likely to discourage handwashing with soap since the water is expected to be taken by children, and has considerable health concerns.

“*When a child is given water that is used to wash hands, they say the child doesn’t take time to grow up*”.

*(36-year-old-woman, FGD)*

For infants, if diapers are soiled, we observed that they were usually folded over and stored inside the house until the next opportunity for washing at the lake; observers saw few instances where hands were washed after handling infant bottoms. At a funeral ceremony attended by an observer, it was discovered that handwashing with soap at such occasions would be viewed as a show of no pity, and they have never seen it happening. Previously, the whole community would not bath if a funeral happened in a show of shared sorrow, a practice they alleged to have waned. A further barrier to hygiene was the limited time to take adequate care of infants and the house due to attention that the fish business necessitates.

“*For smoked fish we spend a lot of time to prevent them from being burnt. So, we spend the whole day just working on the fish nothing else until we are done. After we finish smoking we have time to cook for the children then go to the market in the evening to sell the fish*”.

*(31-year-old female fish processor, IDI)*

#### 3.4.3. Fish Hygiene

We observed that the fishing currently done in the Likulungwa fishery is entirely artisanal and operated by a few individuals. Fishermen chose to sell their catch fresh early in the morning to avoid it going bad. In order to access good quality fresh catches, fish processors camp in nearby rest houses at the trading center to buy and process fish (drying and smoking). Observers saw that hygiene conditions at these processing sheds were severely compromised. Observers had the opportunity to see processing at four different sheds on multiple occasions. Water was drawn from the unprotected wells, and was only used for preparing meals and washing hands before eating; observers did not witness hand washing at any stage during fish handling and processing. Flies, waste, and livestock such as ducks and ducklings were a common sight in these sheds, and were in direct contact with fish and fish products. There was one latrine observed per shed, located approximately ten meters from the fish processing site. None of the latrines had a drop hole cover and they were smelly with flies moving outside and in the latrine. The latrines were not cleaned all the days when the sheds were being observed. The fish processors used the latrines, but some were observed urinating outside the latrine even when the latrine was free. Observers saw that most fish processors kept the processed fish in baskets, with others using plastic buckets; baskets were incapable of being effectively cleaned and disinfected, and processors did not wash the buckets before use. Mosquito nets were used as a surface during the sun drying process by hanging them on a raised platform primarily constructed from local bamboo and reeds. These cloths and surfaces were not cleaned or disinfected before use. The mosquito nets were hanged on a raised platform and were not cleaned before use. On one processing occasion attended by an observer, the same visibly dirty rag used to sort fish was used to wipe the face of the processor in order to remove sweat. From informal conservations, one fish processor laughed saying: *“Hahaha, washing these rags is not important”.*

## 4. Discussion

Overall, our study highlighted the shortfalls in both WASH infrastructure and practice in the marginalized fishing populations, which were largely perpetuated through a lack of resources, skills, and maintenance of social norms.

Our findings highlight acute limitations in access to safe water in this fishing community, and show that drinking water was mainly collected from unsafe sources, often requiring more than 30 min per trip. The WHO and UNICEF classifies no more than 30 min per trip to collect water, as having at least “basic” drinking water services [22]. Overall, these findings underline the great number of people without “basic” access to safe water in this fishing community; and inadvertently the community is exposed to unsafe water which potentially leads to transmission of pathogens. Further, limited access impacted on women’s participation in economic activities. An Ethiopian study showed that poor access to safe drinking water greatly influences the participation of girls and women in education, agricultural production, and other development activities [23]. Evidently, equitable provision of safe water remains a challenge to policy makers, therefore, water supply initiatives should monitor safe water supplies so that water supply actors are furnished with more detailed data about existing inequalities, and can better target marginalised settings such as fishing communities.

We observed that accessibility factors, water quality perceptions, and intended use combined to affect the community’s water source preferences regardless of the source’s inherent risk to health. Findings of this nature are not novel or confined to fishing communities or Malawi, studies in Ghana and Cambodia observed that aesthetic factors influenced water source preferences [24,25]. Direct experience through taste has been found to have a strong role in perceptions of water quality and its relevance ought not be underestimated in consumers’ assessment of drinking water, especially by generating expectations about what good drinking water should taste and look like [26]. Noticeably these practical aspects play an important role in water source preference, and it is important to address existing concerns associated with safe sources through infrastructural improvements, and health promotion initiatives. Similarly, our results support previous findings that water treatment and management promotion must be behaviour centered and context appropriate, taking into consideration the needs and wants of the community it is targeting [24,27,28,29,30,31,32]. Capacity in households and communities to monitor drinking water quality through practical and cost-effective methods, could re-enforce these messages in a more effective manner, as has been achieved with visual behaviour change techniques (e.g., Glo Germ^™^) used elsewhere in Malawi [33,34,35].

Although a large proportion of households were found to have latrines, these were basic and vulnerable to collapse due to topography, inappropriate design, poor construction, and incorrect use [36,37,38]. As such, coastline communities either require high initial investment to minimize collapse, or incur consistent costs associated with reconstruction every 11–13 months [39,40]. Decision makers should therefore ensure that such communities have capacity to construct alternative improved low-cost latrine technologies that suit their environmental conditions. For instance, it is recommended that if the latrine design has a maximum possible life of under ten years, use of alternating double-pit system should be considered, this initiative should be done simultaneously with the promotion of safely managed sanitation [39,40].

We found that latrine sharing was higher (65%) than the national estimate (28%), caused in part by the movement of transient fishermen, processors, and buyers. Although the open defaecation rate was lower (0.8%) than the national average (6%) [41], this was self-reported and therefore prone to bias. The casual approach to handling child faeces, especially of younger children, is consistent with findings from demographic surveys in Malawi and Ethiopia which show that safe disposal of stools mostly increases with children’s age [41,42]. Such existing faeces management practices could predispose the household to faecal-oral infections, as such the community ought to be aware of the dangers associated with improper passage, transport, and disposal of faeces at all ages, including livestock.

Notwithstanding the sanitation within the community, our findings show that sanitation represents a huge challenge among fishermen when doing their work, which is consistent with findings in literature [2,12,43]. The nature of their work—which is mostly done at night and deep into the lake—renders sanitation options to be very restricted. Owing to these conditions, fishermen defaecate and urinate in the lake, this practice potentially increases the risk of transmission of water-related diseases. A related study in Malawi found that fishermen openly defaecate in Lake Malawi when fishing due to the long hours of their work, although the rate of open defaecation among fishermen remains unknown [12]. In addition, potable water is not taken on board the boat, and consequently, fishers rely on lake water for drinking [12]. This practice was recognised within the entire community, and has been normalised as an acceptable practice. The practice is further strengthened with the community-wide belief that fish eat the faeces and the lake self-cleanses. Possibly, such misconceptions have the propensity to further fuel the practice. These findings show that sanitation options are limited in fishermen, as such decision makers ought to ensure that innovative practical approaches are available to fishermen to suit their unique WASH needs.

Coverage of HWFs was lower compared to the national estimate (83%), although availability of soap was higher [41]. However, as the majority of the handwashing facilities in our study were mobile, any tablet of soap available could have been shown thereby amplifying the picture. Worldwide, according to global, regional, and country estimates, in 2015, one in four persons did not have access to a handwashing facility with soap and water on premises [44]. Despite near universal knowledge of importance and benefits of handwashing, our observed findings indicated suboptimal handwashing practices among community members of all ages. These findings mirror the local picture where a previous study found that only 5% washed hands with soap after potential faecal contact occurrences [45]. Anal cleansing was regarded as adequate with no need of further handwashing outside the latrine. However, there are few articles on anal cleansing and how it influences other WASH behaviours, as such, there is a need for further examination of anal cleansing and WASH, particularly in these low income Muslim populations with little infrastructure and access to WASH services. These results indicate that awareness of the context in which community members were practising hand washing and the existing normative, behavioural, and structural barriers that potentially influenced and perpetuated these practices is paramount. It may be inappropriate to require HWF to be installed outside a latrine to appease government standards, if they will not be used. As such, a more context appropriate solution should be sought which is acceptable to both the population and policy makers. In addition, provision of infrastructure to promote handwashing with soap at critical times may be inadequate without concurrent development of effective behaviour promotion approaches, and access to adequate water.

Our findings show that the hygiene practices in fish processing and quality management are not considered during handling, drying, and smoking. Such practices further promote the risk of microbial contamination of fish and fish products, as outlined in previous reports of poor fresh fish handling in Malawi [12,46]. Dried and smoked fish products which are deemed ready to eat are of particular concern, and it is important that fish hygiene should be emphasized in the fish value chain to ensure the safety and value. It is essential that interventions in fish management continuum should be cognizant of catch to consumption, and that hygiene and quality standards should be maintained even for artisanal production. Future interventions must consider training and behaviour change strategies that emphasize adherence of minimum hygiene standards.

Our findings should be interpreted cognizant of the limitation of time, and the range of individuals with whom we engaged. Future studies should consider a specific focus on the practices and behaviours of transient populations in this setting, and food safety practices for specific fish products to help support specific recommendations in those areas.

## 5. Conclusions

We found that fishing communities are underserved and marginalised in terms of WASH infrastructure and practices at both household and commercial (fish production) level. Fishing communities lack basic WASH facilities such as safe water, improved toilets, and handwashing facilities despite their near presence to an important natural resource. Their access to water and sanitation is affected by the topography and hydrogeology. Practices such as water treatment, safe defaecation (especially among fishermen), and overall handwashing are suboptimal in fishing communities, and are further influenced by social and cultural norms. Future interventions in terms of both infrastructure and behaviour change communication should be cognizant of the unique context within these marginalised communities.

## Figures and Tables

**Table 1:** Water access, treatment, and storage practices.

Factor	*N*	Percent (%)	95% CI
**Drinking water source**			
Borehole	68	28.1	22.5–34.2
Unprotected well	171	70.7	64.5–76.3
Surface (Lake) water	3	1.2	0.26–3.6
**Distance to the water source**			
Less than 500 m	116	47.9	41.5–54.4
Between 500 m and 1 km	101	41.7	35.5–48.2
More than 1 km	25	10.3	6.8–14.9
**Roundtrip**			
Less than 30 min	60	24.8	19.5–30.7
Between 30 min and one hour	127	52.5	46.0–58.9
More than one hour	55	22.7	17.6–28.5
**Perception of water being safe for drinking**			
Yes	127	52.5	46.0–58.9
No	114	47.1	40.7–53.6
Do not know	1	0.4	-
**Treat drinking water**			
Yes	96	39.7	33.5–46.1
No	146	60.3	53.9–66.5
**Method of treatment (*n* = 96)**			
Boil	13	13.5	7.4–22.0
Boil and chlorinate	3	3.1	0.7–8.9
Strain through cloth and boil	2	2.1	0.3–7.3
Add chlorine/water guard	74	77.1	67.4–85.1
Strain through a piece of cloth	4	4.2	0.7–12.6
**Frequency of using the method (*n* = 96)**			
Always	11	11.5	5.9–19.6
Frequently	11	11.5	5.9–19.6
Sometimes	74	77.1	67.4–85.1
**Mouth of drinking water storage container (*n* = 224)**			
Narrow	8	3.6	1.6–6.9
Wide	216	96.4	93.1–98.5
**Method of accessing water from the container (*n* = 224)**			
Dipping	217	96.9	93.7–98.7
Pouring	7	3.1	0.1–6.3
**Lid for drinking water storage container (*n* = 224)**			
Lid present	195	87.1	81.9–91.2
Lid not present	29	12.9	8.8–18.1

**Table 2:** Microbiological quality of sampled water points.

Water Sample Source	*E. coli*
	(MPN/100 mL)
Doko 1 Unprotected Well	214
Doko 2 Unprotected Well	61
Doko Main Unprotected Well	71
Mzikiti Borehole	0
Sambammanja School Borehole	1
Doko Borehole	0
Zigumba Centre Unprotected Well	119
Trading Centre Unprotected Well	239
Likulungwa Unprotected Well	11
Lake Malombe	77

**Table 3:** Latrine characteristics.

Factor	*N*	Percent (%)	95% CI
**Presence of Latrine**			
Yes	207	85.5	80.5–89.7
No	35	14.5	10.3–19.5
**Type of Latrine (*n* = 207)**			
Improved latrine	2	1	0.1–3.4
Open pit (Traditional) latrine	205	99	96.6–99.9
**Drop Hole Covers (*n* = 207)**			
Present	116	56	49.0–62.9
Not present	91	44	37.1–51.0
**Latrine Lockable Inside (*n* = 207)**			
Yes	4	1.9	0.5–4.9
No	203	98.1	95.1–99.5
**Latrine Lockable Outside (*n* = 207)**			
Yes	3	1.4	0.3–4.9
No	203	98.1	95.1–99.5
**Materials for Anal Cleansing (*n* = 207)**			
Yes	153	73.9	67.4–79.8
No	54	26.1	20.2–32.6
**Latrine Sharing (*n* = 207)**			
Shared	134	64.7	57.8–71.2
Not Shared	73	35.3	28.8–42.2
